# Development of a New Type of Incisal Table for Prosthetic Articulators

**DOI:** 10.1155/2010/458514

**Published:** 2010-04-22

**Authors:** Alessandro Tasora, Piero Simeone

**Affiliations:** ^1^Dipartimento di Ingegneria Industriale, Università degli Studi di Parma, 43100 Parma, Italy; ^2^V.Bertoloni 1, 00197 Roma, Italy

## Abstract

This study illustrates the effectiveness of an advanced incisal table surface, featuring adjustable curvature, in the sake of more accurate articulator kinematics in anterior teeth reconstruction. Prosthetic articulators, used by dental technicians in reconstructive dentistry, are adjustable instruments that simulate the motion of mastication between dental casts: usually, the forward motion (protrusion) of the mandible is guided by sliding a pin over a flat table in order to recreate those movements when incisal teeth are missing. However, such protrusion is an approximation of the exact motion, since flat incisal tables have a limited set of adjustments. Customized software has been developed in order to simulate the kinematics of articulators in three-dimensional space: animations and measures of the envelope of teeth profiles show the unfeasibility of reconstructing with good approximation the profile of incisive teeth, when a simple ‘flat' incisal table is used. A new incisal table with an adjustable curvature has been proposed, simulated, and built, and computer simulations demonstrated the superior precision of the new design when compared to a conventional articulator which uses a flat incisal table.

## 1. Introduction

**Figure 1:**
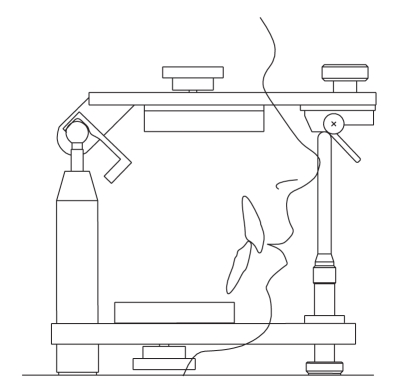
A schematic representation of a dental articulator, as implemented in the interactive editor of the simulation software.

Prosthetic articulators are mechanical devices used in dentistry to assist in the fabrication of prosthodontic restorations and appliances. Casts of the maxillary and mandibular teeth are fixed to two arms that reproduce the motion of the mandible respect to the maxilla (Figure 1). Typically, the upper arm is constrained by conjugate profiles that simulate the mandibular condyles, and by a pin that slides over an inclined table, whose effect is to reproduce the contact between incisal teeth during the protrusion movement [1]. 

Conventional articulators usually allow the adjustment of the inclination of the incisal table but, since the surface in contact with the incisal pin is a flat plane, the end of the pin must follow a simple straight trajectory. This fact advocates the adoption of an incisal table that exploits also an adjustable curvature, so that the original shape of the incisors could be replicated with higher precision. Such outlook is also supported by experimental data: when sliding upper and lower casts of a healthy man, the envelope of motion of the incisal pin engraves a concave hole in a mold block placed on the incisal table [2, 3]. 

This work presents a kinematical analysis of the protrusive movement by means of multibody simulation software [4–7] and demonstrates the advantage of a new incisal table that features a curved contact surface.

Numerical simulations show that the reconstructed profile of the incisors is an inexact approximation of the original shape if conventional flat incisal tables are used. Indeed, the adjustment of the inclination of the plane is enough to obtain a limited set of profiles featuring an almost flat concavity, not adjustable, whereas profiles with more varied curvatures might be desirable, even for the same table pitch.

If curvatures are ignored, as in traditional flat tables, the concavity of the incisal guidance can be underestimated, therefore causing the maxillary incisors to be thicker. This inaccuracy, if modeled prosthesis are not corrected, may affect the occlusion mechanism [8–13]. 

Finally, we propose a compliant device that can be used to implement the concept of the curved incisal table using a small amount of mechanical parts: a thin deformable section is bent up to the desired curvature by turning a knob which bends the surface, hence obtaining the approximation of the needed curvature [14].

## 2. Kinematic Models

A three-dimensional model of the articulator has been implemented in our in-house multibody software *Chrono::Engine*, customized with special features for the analysis of such problems. The model is made of two moving parts, mandible and maxilla, and of two holonomic constraints for anterior and posterior guidance (Figure 1). To obtain the highest precision, dynamic and kinematic analyses are computed using a special method which uses a precise NURBS reproduction of the contact surfaces [15].

The design of the simulated mechanical parts is inspired to an adjustable Arcon-type articulator featuring values for the condylar guide eminence angles, shape of condylar guides, pitch and vertical tilting of the incisal table, and length of the incisal pin. The shape of the teeth can be modified in order to study patients with different anterior guidance. The protrusion can be adjusted via an automated simulation cycle, which runs through 6–8 mm of protrusion imposed by a virtual actuator.

 The reciprocal motion of the two parts (maxillary and mandibular arches) is studied using two different modes

### 2.1. MODE A

**Figure 2:**
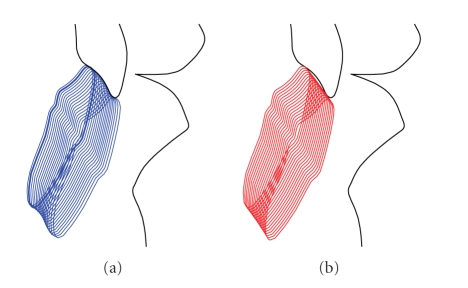
(a) simulation of articulator motion for an 8 mm protrusion, considering an exact contact between incisors belonging to a healthy man (*MODE A*). (b) Envelope of incisors reconstructed using the contact between a conventional flat incisal table and the incisal pin (*MODE B-flat*).

For the posterior guidance, a sliding constraint (curvilinear glyph) represents the condylar guides, while anterior guidance is represented by a sliding constraint between surfaces of maxillary and mandibular incisors. This mode simulates the physiological kinematics of the occlusion; Figure 2(a) shows the superimposition of multiple snapshots of teeth and the relative motion of maxilla and mandible.

### 2.2. MODE B

For the posterior guidance, a sliding constraint (curvilinear glyph) is used for the condylar guides, while anterior guidance is obtained by means of a sliding constraint between the incisal pin and the incisal table. This mode simulates the mechanical behavior of the articulator as constrained by the incisal table, ignoring the effect of the contact between incisors.

This simulation mode can be divided in two further subcases. In *MODE B-flat*, a conventional flat incisal table is used, thus giving approximate results. 

**Figure 3:**
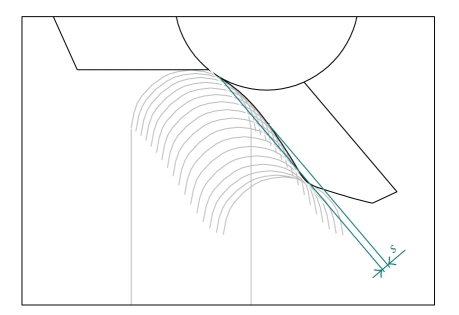
Computer simulations show that the ideal profile of the incisal table should not be flat: the curvature is more noticeable near the position of intercuspal occlusion.

In *MODE B-curved, ideal*, an exact incisal table is used, obtained as the envelope of the hemisphere at the apex of the incisal pin during a simulation performed with *MODE A*. The conjugate profile of the incisal pin is concave: the surface has a downward curvature, denoted with the *s* symbol in Figure 3.

## 3. Simulation Results

Simulations for *MODE A* show that the incisal pin, driven by contact between true incisors and motion of condyles in the glenoidal fossa, describes a trajectory that, in general, exploits some kind of variable curvature. 

The error caused by the adoption of a conventional flat incisal table can be measured by comparing results coming from the ideal *MODE A* to simulations performed in *MODE B-flat*. In the latter case, the top end of the incisal pin is constrained on the flat surface of the incisal table: despite all the admissible settings of alignment for a standard incisal table, it will be difficult to obtain a close approximation of the profile of the original incisal guide, represented by true contact between healthy teeth. In fact, if a flat table is rotated in order to obtain a good approximation of the incisal guide for the initial part, the thickness of the maxillary incisors will be underestimated. On the other hand, if the table is rotated so that the last part is approximated more accurately (Figure 2(b)), the thickness of the maxillary incisors will be overestimated in the middle part. Thickening error can range in the 0.00 *÷* 0.80 mm interval. 

Moreover, dynamical simulations show that, even if the amount of the thickening may be small, at the point of contact the direction of surface normals may be noticeably different from the one of the ideal case, and the effect would be a different direction of reaction forces. Such error in force orientation may range in the ±20° interval: this could cause negative side effects from the gnatological point of view.

**Figure 4:**
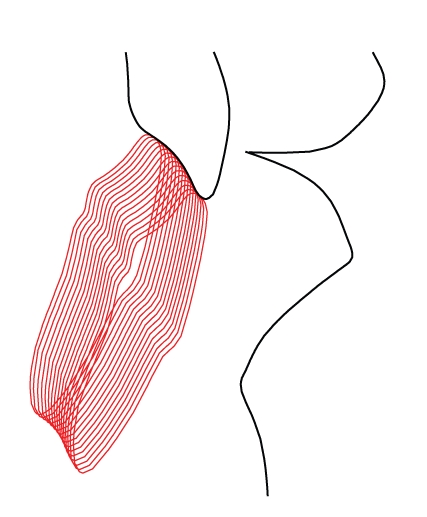
Envelope of the incisors when motion is constrained by the contact between the incisal pin and the ideal incisal table (*MODE B-curved, ideal*). The original teeth shape is accurately rebuilt.

Various simulations have been automatically performed to investigate the main parameters which may affect the curvature of the incisal table. Hence, a range of tests has been simulated for different values of eminence angles, different shapes of condylar guides, and different teeth profiles. For all these cases, simulations have been performed in *MODE A*, hence by enforcing contact between real incisors and contact in the curvilinear condylar guides. In this way, the ideal profile of the incisal table is obtained as the envelope of the incisal pin (Figures 3 and 4).

The profile of the ‘‘ideal” incisal tables has been analyzed in terms of curvature, so that the effects of the parameters have been recognized and studied. From the abovementioned analyses, the most relevant results can be summed up as follows.

The curvature radius can change depending to many factors. In general, given a spherical incisal pin with radius *R*_*a*_, and denoting the radius of the curvature of the trajectory of the center of the pin with *R*_*c*_, the radius of the table is *R*_*p*_ = *R*_*a*_ + *R*_*c*_. Kinematic analyses show that *R*_*c*_ can be as low as 2 mm. In all cases, the curvature is more noticeable in the first part of the protrusion and progressively decreases during the protrusion up to a flex (null curvature) about 7-8 mm away from the position of centric occlusion. 

Changes in the shape of the condylar guides have small effects either on the average inclination of the ideal incisal table or on its curvature; on the other hand, changes in the shape of the incisal guide have noticeable consequences on the shape of the ideal incisal table. There is almost a linear relation between the average inclination of the ideal incisal table and the average inclination of the incisal guidance of real teeth. Also, for growing values of incisal guidance curvature there are growing values of incisal table curvature.

## 4. New Articulator Design

**Figure 5:**
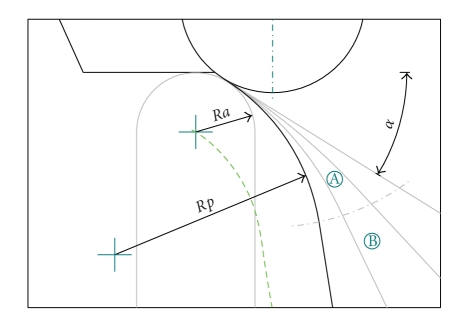
Simplified case: a compliant incisal table approximates the ideal shape by changing the uniform curvature of a flexible section ‘‘A”, while section ‘‘B” is rigid and straight.

Given the requirement of a simple and intuitive method to adjust the articulator, a single parameter, namely, the curvature, could be used to approximate the ideal anterior guidance using a bendable table (Figure 5). This practical implementation of curved incisal table exploits two zones: a flexible ‘‘A” section with uniform curvature (whose radius *R_p_* may be adjusted up to *R*_*p*_ = ∞ depending on user needs) and a straight ‘‘B” section, without curvature. Moreover, the entire profile can be rotated forward/backward by an angle *α*. The case *R*_*p*_ = ∞ would correspond to null curvature, as in the case of flat tables for conventional articulators. 

**Figure 6:**
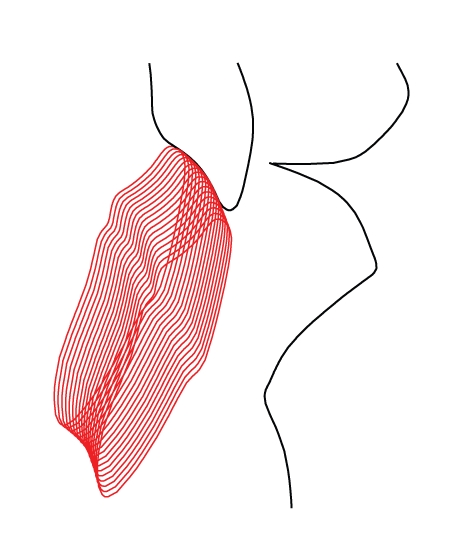
Simplified case: envelope of the incisors when motion is constrained by contact between the incisal pin and the novel incisal table featuring uniform-curvature.

In order to test the precision of this kind of profile, heretofore defined ‘‘simplified” because it can be easily implemented by means of a mechanical device featuring adjustable curvature, the multibody software performed simulations (Figure 6) which compared the results of this mode with the results corresponding to the ideal case. The approximation that has been introduced by switching from an ideal incisal guide, featuring the exact nonuniform curvature, to the simplified profile, is so low that it can be neglected: from our simulations, the approximation is usually lower than 0.1 mm.

**Figure 7:**
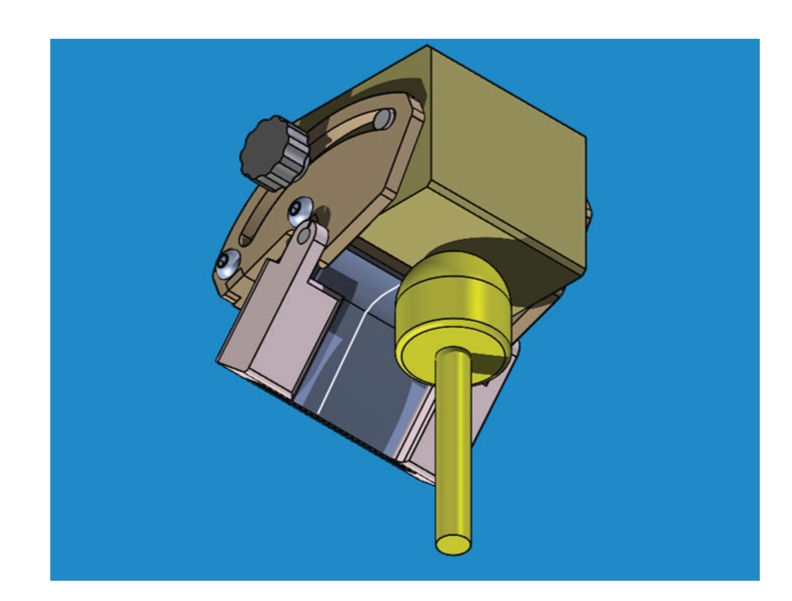
A preliminary, simplified hypothesis for the compliant incisal table, to be mounted on a typical commercial articulator. Curvature can be adjusted by turning a screw and bending the table.

**Figure 8:**
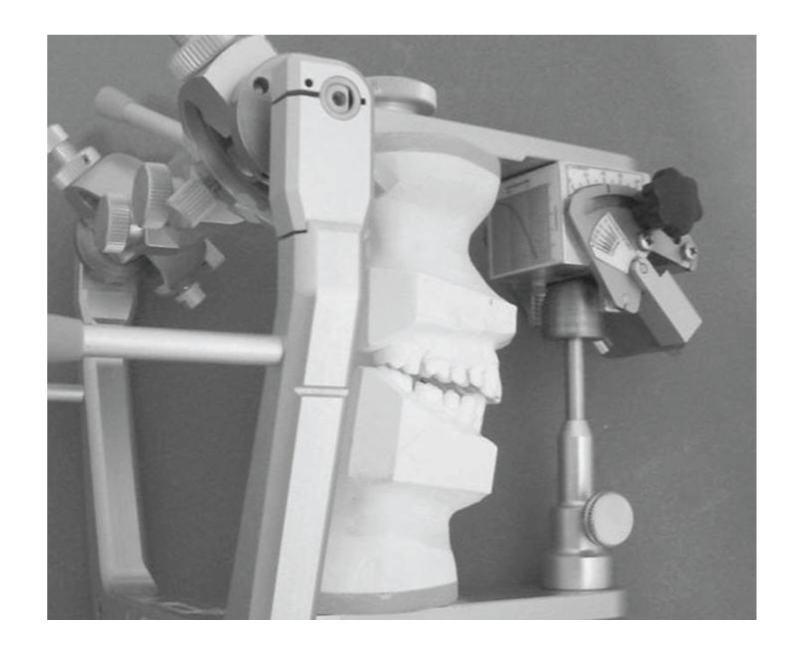
The prototype of the novel incisal table, mounted on a commercial articulator.

Figures 7 and 8 show a preliminary design for an incisal plate that can be adjusted in terms of curvature. The variable curvature, on the first 7 mm of contact, is obtained by bending a metallic ‘‘compliant mechanism”, where the surface that must be curved can be made of a thin deformable layer exploiting selective flexibility. The user could turn a knob until the desired curvature is obtained; yet we remark that this device can still work in a null-curvature configuration: in such a case, the effect would be identical to a conventional articulator.

The compliant surface is a critical part of the design because it must withstand strong curvatures without breaking or plasticizing. Since polymeric materials can feature early plasticization, steel foils have been preferred. However, curvature of a steel foil is limited by the yield strength *σ*_*y*_: to avoid plasticizing, the maximum bending stress, in the case of maximum curvature, must be *σ*_max_ < *σ*_*y*_. According to the Euler-Bernoulli theory for pure bending, given a foil with thickness *b* and moment of inertia *J *subject to a moment *M *about neutral axis, we have *σ*_max_ = *M* *b*(2*J*)^−1^ and curvature *γ* = *R*_*p*_^−1^ = *M*(*E* *J*)^−1^. With few substitutions, one gets

(1) $$R_{p}>Eb{\left(2\sigma_{y}\right)}^{-1}.$$

In order to keep the *R*_*p*_ limit as low as possible, we adopt high-strength laminated steel with Young modulus *E* = 200 GPa and *σ*_*y*_ = 900 MPa. Since *R*_*p*_ = *R*_*a*_ + *R*_*c*_, where the curvature *R*_*c*_ of the trajectory of the center of pin must be as low as 4 mm to approximate the discussed kinematic requirements, we used a foil thickness *b* = 0.1 mm and a pin radius *R*_*a*_ = 10 mm. Larger *R*_*a*_ values would allow even larger *b* thickness still satisfying (1), but at a cost of making a large assembly whose usage might look uncomfortable and atypical to the user (most conventional articulators have small pin radii, about 2-3 mm). On the other hand, lower values of *b* would allow even smaller *R*_*p*_ values (thus lower *R*_*a*_ and a more compact pin), but the surface would be too delicate. Moreover, we used a pack of 20 layered foils for a total thickness of the incisal table of *b*_tot_ = 20  *b* = 2  mm; this avoids that the pressure of the pin over the table could cause damages such as wear or unwanted deformations.

## 5. Conclusion

Multibody simulations made possible to obtain the ideal shape of the incisal table by computing the conjugate profile of the incisal pin during the simulation of an ideal, physiological protrusion. Software analysis showed the superior precision of a prosthetic articulator featuring an adjustable curvature of the incisal table, when compared to a conventional articulator with a simple flat incisal table. A prototype of table with adjustable curvature, where the user can modify the profile of the table by acting on a knob, is currently under testing and clinical results will follow.
